# Genome-wide association study of intraocular pressure identifies the GLCCI1/ICA1 region as a glaucoma susceptibility locus

**DOI:** 10.1093/hmg/ddt293

**Published:** 2013-07-07

**Authors:** The Blue Mountains Eye Study (BMES) and The Wellcome Trust Case Control Consortium 2 (WTCCC2), Amy Strange, Céline Bellenguez, Xueling Sim, Robert Luben, Pirro G. Hysi, Wishal D. Ramdas, Leonieke M.E. van Koolwijk, Colin Freeman, Matti Pirinen, Zhan Su, Gavin Band, Richard Pearson, Damjan Vukcevic, Cordelia Langford, Panos Deloukas, Sarah Hunt, Emma Gray, Serge Dronov, Simon C. Potter, Avazeh Tashakkori-Ghanbaria, Sarah Edkins, Suzannah J. Bumpstead, Jenefer M. Blackwell, Elvira Bramon, Matthew A. Brown, Juan P. Casas, Aiden Corvin, Audrey Duncanson, Janusz A.Z. Jankowski, Hugh S. Markus, Christopher G. Mathew, Colin N.A. Palmer, Robert Plomin, Anna Rautanen, Stephen J. Sawcer, Richard C. Trembath, Nicholas W. Wood, Ines Barroso, Leena Peltonen, Paul Healey, Peter McGuffin, Fotis Topouzis, Caroline C.W. Klaver, Cornelia M. van Duijn, David A. Mackey, Terri L. Young, Christopher J. Hammond, Kay-Tee Khaw, Nick Wareham, Jie Jin Wang, Tien Y. Wong, Paul J. Foster, Paul Mitchell, Chris C.A. Spencer, Peter Donnelly, Ananth C. Viswanathan

## Abstract

To discover quantitative trait loci for intraocular pressure, a major risk factor for glaucoma and the only modifiable one, we performed a genome-wide association study on a discovery cohort of 2175 individuals from Sydney, Australia. We found a novel association between intraocular pressure and a common variant at 7p21 near to GLCCI1 and ICA1. The findings in this region were confirmed through two UK replication cohorts totalling 4866 individuals (rs59072263, *P*_combined_ = 1.10 × 10^−8^). A copy of the G allele at this SNP is associated with an increase in mean IOP of 0.45 mmHg (95%CI = 0.30–0.61 mmHg). These results lend support to the implication of vesicle trafficking and glucocorticoid inducibility pathways in the determination of intraocular pressure and in the pathogenesis of primary open-angle glaucoma.

## INTRODUCTION

Elevated intraocular pressure (IOP) is a major risk factor for the development and progression of glaucoma, which is the commonest cause of irreversible blindness worldwide (1). Globally, 60 million people are affected of whom 8.4 million are blind in both eyes (2). Before this end stage there is a greater risk of falls (3), fractures (4) and motor vehicle collisions (5).

The link between high IOP and glaucoma is clearly seen when there is a readily identifiable cause for raised IOP such as trauma, inflammation, dysgenesis or obstruction of the aqueous humour drainage system in the trabecular meshwork of the iridocorneal angle by iris tissue, pigment or other deposits (2). It is also evident even when there is no clear cause or obstruction of the iridocorneal angle. Glaucoma occurring under these latter conditions is known as primary open-angle glaucoma (POAG) and is the most prevalent form of glaucoma, causing the greatest burden of disease (6). The risk of developing POAG is approximately five times higher in subjects with IOPs above the population 95th centile than in subjects with lower IOPs (7); the higher the IOP at screening, the greater the risk of POAG (8). Both mean and maximum IOP have been reported as closely associated with visual field deterioration (8–15) and optic nerve damage (16). In a group of subjects affected by POAG despite untreated IOPs below the population 95th centile (‘normal pressure’ glaucoma), worse visual field damage was found in the eye with the higher IOP (17). Furthermore, IOP is currently the only modifiable risk factor for POAG. Therapeutic lowering of intraocular pressure reduces the risk of developing glaucoma (18) and retards disease progression (19–21). In addition to inherent interest, and its role as a modifiable trait on the causal path to disease, the known heterogeneity of POAG means that a focus on the genetic basis of IOP may be more powerful than studies of POAG directly (22,23).

Heritability estimates for adult IOP range from 0.29 to 0.62 (24–28). To date, two regions have been reported to be robustly associated with IOP as a result of linkage analyses, one at 10q22 from a Tasmanian glaucoma pedigree (29) and one at 5q22 in a West African cohort (30). A recent Dutch genome-wide association study (GWAS) for IOP (31) also found two associated loci, one of which (*TMCO1*) overlaps with a previously published POAG GWAS locus (32).

Further elucidation of the genetic basis of IOP would aid in understanding the biology of a major blinding disease (POAG), in risk stratification for development and progression of the disease and in providing therapeutic targets. In order to identify genomic variants that contribute to the determination of IOP, we performed a GWAS as a component of the Wellcome Trust Case Control Consortium 2 (WTCCC2) (http://www.wtccc.org.uk/ccc2/).

## RESULTS

A total of 2765 discovery samples from the Blue Mountains Eye Study (BMES) were genotyped on the Illumina 660W-Quad array at the Wellcome Trust Sanger Institute (WTSI). Genotype imputation was performed in all 2302 individuals passing genotype quality control (QC) using IMPUTE2 (33) and the 1000 Genomes reference panel (34) (see Materials and Methods and Supplementary material online). SNPs with low imputation quality (info < 0.7) (35) were excluded, resulting in 6 235 970 SNPs on chromosomes 1–22 which passed QC.

For each individual, the mean IOP between their two eyes was calculated. If an individual had undergone surgery or treatment to an eye, data from that eye were excluded. If the difference in IOP between an individual's two eyes was >10 mmHg, the individual was excluded. Eight outlying individuals whose measurements did not conform to a normal distribution were also removed (see Materials and Methods). After genotype and phenotype QC, a total of 2175 samples were available for analysis.

**Figure 1.**
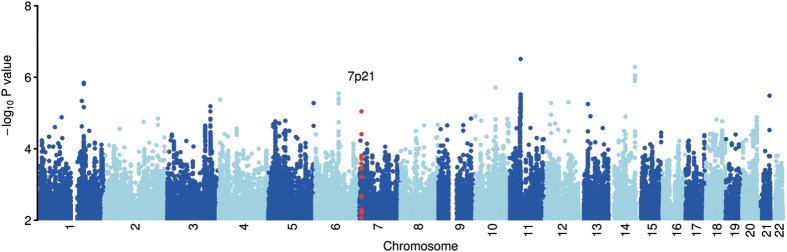
Plot of genome-wide association results after fitting the additive model in SNPTEST. Genome-wide association results in the discovery data at 6 235 970 SNPs (5 718 276 imputed, 517 694 genotyped), each represented as a point on the plot. Chromosomes are coloured dark blue and light blue alternatively, as labelled on the X-axis. The –log10 (*P*-value) is shown on the Y-axis. The locus identified in this study as being associated with intraocular pressure is highlighted in red and labelled by chromosomal region.

A linear regression fitting an additive model was performed in SNPTEST (https://mathgen.stats.ox.ac.uk/genetics_software/snptest/snptest.html), where genotype, accounting for imputation uncertainty, was set as the explanatory variable and mean IOP as the response variable (Fig. 1). Age and sex were included as continuous and categorical covariates, respectively. This analysis will be referred to as the primary scan. The genomic inflation statistic (λ) was 1.01 (Supplementary Material, Fig. S1), suggesting that population structure was not a major problem in our discovery analyses.

Replication of the primary scan was attempted at 31 SNPs covering 17 regions (Supplementary Material online, Table S1 and see Materials and Methods), of which two were regions previously found to be associated with IOP (31). Two platforms were used for the first phase of replication; genotypes for four SNPs were taken from the custom built Immunochip (36) (see Materials and Methods), and genotypes for the other 27 SNPs were obtained from a Sequenom plex. Results from discovery and replication were combined using a fixed effects meta-analysis by averaging the estimated effect size parameter across the data sets, weighting by the inverse of the variance in the estimates.

For the first phase of replication, we used individuals from the European Prospective Investigation into Cancer (EPIC) Norfolk cohort (see Materials and Methods). Following QC, data were available for 2833 individuals genotyped on Sequenom and 2493 individuals genotyped on Immunochip (all with age, sex and mean IOP data) with 2461 individuals overlapping between the Sequenom and Immunochip replication cohorts.

The results from our data at SNPs previously found to be associated with glaucoma (32,37–39) or IOP (31) are shown in Table 1. Notably, the discovery data in our study contributed to the replication phase in the IOP GWAS (31). An SNP in *TMCO1* (rs7555523), which has been associated with IOP (31) showed evidence for association with mean IOP in both our discovery (*P* = 0.0303) and replication data (*P* = 1.70 × 10^−3^). At the other region previously associated with IOP (31), on chromosome 17p13 (rs11656696), there was no evidence for association in our discovery data (*P* = 0.548), whereas there was evidence for association in our replication data (*P* = 0.016). One of the SNPs previously associated with POAG (39) was also associated with mean IOP in our data, (rs4236601, *P* = 1.46 × 10^−3^). The remaining previously associated SNPs were not associated (*P* < 0.1) with IOP in our discovery data.

**Table 1.** Evidence for association in the discovery data at SNPs previously found to be associated with glaucoma or IOP

Chr	rsID	Position	Gene region	Reported risk allele	BMES discovery *P*-value (risk allele)	EPIC replication *P*-value (risk allele)	Phenotype	Reference
1	rs4656461	163953829	TMCO1	G	0.309 (G)	—	Glaucoma	(32)
1	rs7555523	163985603	TMCO1	C	0.030 (C)	1.70 × 10^−3^ (C)	IOP	(31)
2	rs3213787	45500328	SRBD1	A	0.130 (A)	—	Glaucoma	(37)
6	rs735860	53231077	ELOVL5	C	0.796 (T)	—	Glaucoma	(37)
7	rs4236601	115949965	CAV1, CAV2	A	1.46 × 10^−3^ (A)	—	Glaucoma (POAG)	(39)
9	rs4977756	22058652	CDKN2B-AS1	A	0.623 (A)	0.590 (A)	Glaucoma	(32)
15	rs3825942	72006635	LOXL1	G	0.553 (A)	—	Glaucoma (exfoliation)	(38)
17	rs11656696	9974404	GAS7	G	0.548 (G)	0.016 (G)	IOP	(31)

‘—’ data not available.

**Figure 2.**
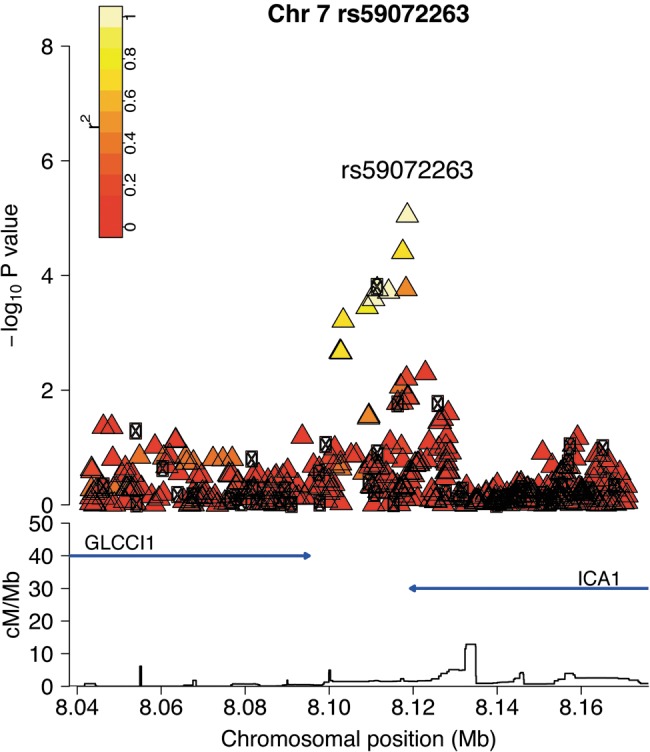
Regional association plot of the locus found to be associated with intraocular pressure. The –log10(*P*-values) for the additive model SNP association in SNPTEST are shown on the upper part of the plot. SNPs are coloured based on their *r*^2^ with the labelled SNP, *r*^2^ calculated in the 1958 Birth Cohort data. Circles represent directly genotyped SNPs, and triangles represent imputed SNPs. The bottom part of the plot shows the fine scale recombination rate estimated from individuals in the HapMap panel, with genes marked by horizontal blue lines.

Following first phase replication, one of the newly identified regions reached a *P*_combined_ <5 × 10^−8^. This was on chromosome 7p21 and the top SNP (rs59072263, *P*_combined_ = 2.32 × 10^−8^) is between the genes *ICA1* and *GLCCI1* (Fig. 2 and Supplementary Material, Fig. S2). Genotypes at this SNP were imputed in the discovery data and directly genotyped in the replication. To check the reliability of the imputed calls, a subset of 214 discovery samples were directly genotyped at rs59072263. The concordance between the imputed and genotyped calls was 0.96 (eight out of 214 discordant calls) and the correlation was 0.87, suggesting the imputation at this locus is acceptable.

No evidence for replication at *P* < 0.05 was found at the other 14 regions (Supplementary Material, Table S1). Two SNPs at 11p12 (rs10430986 and rs12222492) showed suggestive evidence for association in the same direction as the discovery data (*P* = 0.07, *P* = 0.11), although data were not available for the top SNP in the region (11-41102934). This locus may be worth additional investigation.

A second round of replication of the 7p21 association was next attempted in 2033 individuals from TwinsUK (40), in which the genotypes at rs59072263 were imputed. The SNP had a one-sided *P*-value of 0.053 assuming the same risk allele as the discovery, giving combined evidence across the three cohorts of *P* = 1.10 × 10^−8^ (Supplementary Material, Fig. S3), where the G allele is associated with an IOP increase of 0.45 mmHg (95% CI = 0.30–0.61 mmHg).

Replication of the signal of association at 7p21 was also assessed in the large Dutch meta-analysis (31) of IOP comprising 9680 individuals of European ancestry. The associated SNP rs59072263 was not genotyped in this study, but the best available tag (rs6959703; genotype correlation, *r*^2^ = 0.61) showed a one-sided *P*-value = 0.017. Imputation of genotypes by the Rotterdam group at rs59072263, using MACH (http://www.sph.umich.edu/csg/abecasis/MACH/index.html) and the 1000 Genomes data, was also attempted but showed weaker evidence of association (one-sided *P*-value = 0.178), However, unlike the discovery data and TwinsUK data, which had imputation accuracy (‘IMPUTE info’ and ‘MACH r^2^’, respectively) of 0.95 and 0.89 at rs59072263, the largest component of the Dutch meta-analysis, comprising almost two-thirds of the study, had MACH *r*^2^ < 0.75. Therefore, particularly in light of the clear replication at the best genotyped tag SNP, the lack of replication in the imputed data at *P* < 0.05 may be due in part to the loss of statistical power which results from the increased noise (imputation uncertainty) in the inferred genotypes (41). We note that the risk allele is the same in all three replication cohorts.

We also undertook a pathway analysis of SNPs with *P* < 10^−4^ (see Materials and Methods), but this failed to pinpoint any pathways with a compelling biological connection to IOP biology.

## DISCUSSION

We have thus identified a novel association between a region on chromosome 7p21 and mean IOP. Since IOP is on the causal pathway to glaucoma, this association is also relevant for susceptibility to glaucoma. The most associated SNP in this region is flanked by two genes, *ICA1* and *GLCCI1*, both of which have been shown to be expressed in the human eye (information from the EMBL-EBI website, see http://www.ebi.ac.uk/gxa/). *ICA1* encodes a protein involved in the regulation of secretory vesicle trafficking (42) and has been implicated as an auto-antigen in insulin-dependent diabetes mellitus (43) and primary Sjögren's syndrome (44). Vesicular metabolism pathways have previously been ascribed a role in the pathobiology of POAG: a case–control GWAS of POAG found associations with the *CAV1* and *CAV2* genes which control vesicle transport in transcytosis mechanisms (39). The latter mediate drainage of aqueous humour from the eye and it is recognized that increased IOP results from a failure of drainage rather than an overproduction of aqueous humour.

The *GLCCI1* gene has been ascribed a role in the sensitivity of various tissues to glucocorticoids, initially thymoma cell lines (45) and recently in the degree of response to glucocorticoid therapy in asthma (46). The variant associated with glucocorticoid therapy in asthma (46) was not associated with IOP in our discovery (*P* = 0.8), and was not in strong linkage disequilibrium (LD) with the IOP associated variant we identified (*r*^2^ = 0.114 in the 1000 Genomes data). Topical glucocorticoid therapy causes raised IOP in susceptible individuals (47) and the likelihood of such an IOP response is greater in POAG patients, POAG suspects and first-degree relatives of POAG patients (48). The BMES has previously reported an association between use of inhaled corticosteroids and a finding of elevated IOP or glaucoma in subjects with a glaucoma family history (49). Furthermore, patients with POAG have elevated blood (50–54) and aqueous humour (52) levels of the endogenous glucocorticoid cortisol compared with age-matched normal subjects. The first glaucoma gene identified, *MYOC* (55), was a glaucoma candidate gene because of its glucocorticoid inducibility (56). It is thus plausible that GLCCI1 may influence IOP via the response to endogenous cortisol.

The effect size of the novel associated SNP we report (Table 2) is substantially higher than that of previously reported SNPs for IOP (beta = 0.45 for rs59072263 when compared with 0.19 for rs11656696 and 0.28 for rs7555523). We can get some indication of the possible consequences of the SNP on POAG, based on its measured effect for IOP, from population studies which relate changes in IOP to POAG risk. This approach suggests that each copy of the G allele at rs59072263 gives an expected odds ratio for POAG of 1.08 (57) and, in untreated glaucoma (in our discovery cohort, in common with other cohorts from the developed world, over 50% of glaucoma was undiagnosed at initial ascertainment) (58), each copy of the allele gives an extra 6% likelihood of significant deterioration in vision (59). The novel associated SNP has a higher risk allele frequency (RAF) than previously reported SNPs (RAF = 0.88 for rs59072263 when compared with 0.58 for rs11656696 and 0.12 for rs7555523) so a higher proportion of our cohort will carry at least one potentially deleterious allele.

**Table 2.** Evidence for association with mean IOP at the 7q21 locus

Chr	Position	rsID	Risk allele	RAF	Discovery (BMES)	Replication (EPIC)	Replication (TWINSUK)	Meta
					*P*-value	*P*-value	*P*-value	*P*-value
					Beta	Beta	Beta	Beta
					SE	SE	SE	SE
7p21	8118592	rs59072263	G	0.88	9.02 × 10^−6^	4.01 × 10^−4^	0.106	1.10 × 10^−8^
					0.61	0.42	0.28	0.45
								
								
					0.136	0.12	0.17	0.08

RAF, risk allele frequency.

In summary, we have performed a GWAS which has newly identified a region at 7p21 associated with IOP; the region is between the *ICA1* and *GLCCI1* genes, both of which may plausibly be invoked in the determination of IOP.

## MATERIALS AND METHODS

### Ethics statement

The Blue Mountains Eye Study received ethical approval by the Western Sydney Area Health Service Human Ethics Committee in 1991. Written, informed consent was obtained from all participants. The EPIC-Norfolk study received approval from the East Norfolk and Waveney Research Governance Committee on 08/03/06 and from the Norfolk1 Research Ethics Committee on 06/02/06.

### Discovery samples

The IOP data from the discovery cohort were gathered as part of the Blue Mountains Eye Study (BMES), a survey of vision and common eye diseases in the Blue Mountains region west of Sydney, Australia. The study was approved by the Western Sydney Area Health Service Human Ethics Committee. Written, informed consent was obtained from all participants. The population has been described in detail in a previous report (60). To summarize, a door-to-door census of the study region was carried out based on maps from the Australian Bureau of Statistics. All permanent non-institutionalized residents with birthdates before January 1, 1943 were invited to attend for eye examination. Of the 4433 eligible individuals, 3654 (82.4%) were examined. If the 278 individuals who had died or moved away from the area are excluded, this translates to an 87.9% response rate, which compares well with most population-based research in glaucoma (61–63). Subjects attending the examination underwent IOP measurement by applanation tonometry using a Goldmann applanation tonometer (Haag-Streit, Bern, Switzerland). Goldmann applanation tonometry is the international standard for accurate determination of IOP in ophthalmic clinical practice. Tonometry could not be performed in 12 subjects (0.3%). In a further nine uniocular subjects, IOP could only be measured unilaterally, in such cases, the value for that single eye was used. Data from subjects receiving past or present treatment designed to lower IOP were excluded from the analysis.

### Replication samples

The European Prospective Investigation into Cancer (EPIC) was conceived as a pan-European study of the genetic and environmental determinants of cancer (64). The EPIC-Norfolk cohort comprised ∼25 000 men and women aged 40–79 recruited between 1993 and 1997 and was predominantly white, with a mixture of urban–rural residence, socioeconomic standards and educational achievements reflecting the population of the county of Norfolk. From the outset, data collection was expanded to include extensive lifestyle and biological data to enable a broader longitudinal study of the determinants of health and disease (65). A third health examination was initiated in 2006 with the purpose of assessing objectively various physical, cognitive and ocular characteristics of participants now aged 48–91 years. The third health examination was reviewed and approved by the East Norfolk and Waverney NHS Research Governance Committee (2005EC07L) and the Norfolk Research Ethics Committee (05/Q0101/191). The work was performed in accordance with the principles of the Declaration of Helsinki. IOP was measured three times in each eye using a noncontact Ocular Response Analyser (ORA, Reichert Inc., Buffalo, NY, USA) to generate the Goldmann Correlated IOP (IOPg) which the manufacturers have calibrated with Goldmann applanation tonometry. Data from subjects receiving past or present treatment designed to lower IOP were excluded from the analysis.

The TwinsUK data were obtained from a panel of individuals who were recruited from the UK Adult Twin Registry (40) based at St Thomas' Hospital, London. The subjects were twin volunteers from the general population. Subjects were recruited for studies other than eye studies, and subsequently asked to attend for an eye examination. All subjects provided informed consent, and the study was reviewed by the Local Research Ethics Committee. The methods adopted in this study adhered to the tenets of the Declaration of Helsinki Principles. IOP measurement was performed with the Goldmann applanation tonometer and the ORA (IOPg). Data from subjects receiving past or present treatment designed to lower IOP were excluded from the analysis.

### DNA preparation

DNA was extracted from whole blood. Quality was validated using the Sequenom iPLEX assay designed to genotype four gender SNPs and 26 SNPs present on the Illumina Beadchips. DNA concentrations were quantified using a PicoGreen assay (Invitrogen) and an aliquot assayed by agarose gel electrophoresis. A DNA sample was considered to pass quality control if the DNA concentration was ≥50 ng/μl, the DNA was not degraded, the gender assignment from the iPLEX assay matched that provided in the patient data manifest and genotypes were obtained for at least two-thirds of the SNPs on the iPLEX.

### Genotyping

Discovery samples were genotyped at the Sanger Institute on the Illumina Infinium platform using the Human660W-Quad, a custom chip designed by WTCCC2 and comprising Human550 supplemented with 60 000 additional probes that were intended to allow the genotyping of common CNVs from the Structural Variation Consortium (66). Replication genotyping for the UK EPIC cohort was carried out at the Sanger Institute using the Illumina Immunochip, a custom chip designed by the Immunochip Consortium and WTCCC2, comprising 196 524 SNPs. For both chips, bead-intensity data were processed and normalized for each sample in BeadStudio; data for successfully genotyped samples were extracted and genotypes called using Illuminus. Replication genotyping on the Sequenom plex was also carried out at the Sanger Institute. Imputation was performed with reference to HapMap release 22 CEU by using IMPUTE2 (33). Genotyping for the TwinsUK cohort was carried out by using Illumina (San Diego, CA, USA) genotyping platforms; the Human Hap 300k Duo and Human Hap610 Quad array. All SNPs passed quality control criteria (Hardy–Weinberg equilibrium *P* > 0.001, minor allele frequency of at least 0.04, genotyping success rate for the SNP at least 95%). Imputation was performed with reference to the 1000 Genomes panel using minimac (http://genome.sph.umich.edu/wiki/Minimac) and association analysis was performed using mach2qtl.

### Quality control

SNPs were excluded if the Fisher information for the allele frequency was not close to unity (information <0.98) or if the minor allele frequency (MAF) was very low (defined as <0.01%), or for extreme departures from Hardy–Weinberg equilibrium (HWE *P*-value <10^−20^). Full details of the quality control methods employed in the Wellcome Trust Case Control Consortium 2 (WTCCC2) have been published elsewhere (67). Also see Supplementary material online, SNP Imputation.

For quality control of samples, a Bayesian clustering method was used to infer and exclude outlying individuals on the basis of ancestry, call rate, heterozygosity and signal intensity (67). To remove signal intensity outliers observed for raw intensity data, the difference between the A channel intensity and the B channel intensity was averaged over all SNPs on autosomes for each sample. A similar approach was used taking intensity measures from the A channel on the non-pseudo autosomal X chromosomes to identify outliers and infer gender. Samples were removed if their inferred gender was discordant with the recorded gender after cross-checking with original database entries, or if <90% of the SNPs typed by Sequenom on entry to sample handling (discussed earlier) agreed with the genome-wide data. To obtain a set of putatively unrelated individuals, we estimated genome-wide identity by descent (IBD) given identity by state (IBS) for all pairs of individuals using a Hidden Markov Model. One of each pair of related individuals where zero alleles shared IBD (IBD0) was <95% was removed in the initial association analysis, excluding the member of the pair with the lowest call rate. Where unexpected duplicates were identified, both of the identical samples were removed to avoid incorrect phenotype assignment. Ancestry outliers were removed by projecting the WTCCC2 individuals on to the first two principal components of a PCA of the CEU, YRI and JPT/CHB HapMap individuals. Samples were excluded based on evidence of non-European ancestry.

A total of 463 individuals were excluded from the discovery cohort following these initial quality control checks.

### 1000 Genomes imputation

We used 120 CEU phased haplotypes from the 1000 Genomes project (34) June 2010 release for the haploid reference panel. After imputation, a total of 7 642 395 SNPs were available for analysis, of which 712 4701 were imputed. SNPs were filtered using an information threshold of 0.7, an allele frequency threshold of 0.1% and Hardy–Weinberg filter of *P* < 10^−20^ giving a final total of 6 235 970 SNPs for analysis (5 718 276 imputed, 517 694 genotyped).

### Pathway analysis

We undertook a pathway analysis using the approach reported previously in Sawcer *et al.* (68). Briefly, we took all SNPs with *P* < 10^−4^ in the discovery data and we used these to define association regions (with at least one such SNP) and compiled a list of all genes in these regions. This list is matched to the Gene Ontology (GO) pathway database. A Fisher’s exact test is used to give a *P*-value for each pathway.

There were 513 genes in our associated regions of which 394 were in the GO database. The Fisher’s exact test highlighted 10 pathways with *P* < 10^−4^ namely: anchored to membrane; monooxygenase activity; *N,N*-dimethylaniline monooxygenase activity; glucuronosyltransferase activity; alkaline phosphatase activity; phospholipid scramblase activity; xenobiotic metabolic process; response to xenobiotic stimulus; cellular response to xenobiotic stimulus; phospholipid scrambling.

Of the pathways identified, those of potentially most interest from a mechanistic viewpoint are ‘phospholipid scramblase activity’ and ‘phospholipid scrambling’. This metabolic activity is a key part of lipid translocation, which drives endo- and exocytosis of microvesicles (69,70). It is thus plausible that this metabolic activity is important in the transcytotic movement of aqueous humour across the trabecular meshwork which is a key determinant of IOP as already mentioned in the manuscript.

We also implemented a number of slight variations on this analysis as in Sawcer *et al.* (68), for example, only including the nearest gene to an associated SNP. These alternative analyses also failed to pinpoint any pathways with a natural biological connection to IOP biology.

### Phenotype

Mean IOP was regressed against age and sex and all eight samples with residuals >10 were removed from the primary analysis. From the replication analysis, 21 individuals with residuals >10 were removed. In the primary analysis, these phenotype outliers were excluded because they can lead to false positive associations. In order to check that we were not missing any true associations by excluding outliers, the analysis was repeated with inclusion of the phenotypic outliers, and a small number of regions (4) were chosen for replication based on this scan. One region for replication was also taken from the analysis fitting a general model with residual outliers included.

Regions were shortlisted for replication if they had at least one SNP with *P* < 10^−5^ in the primary scan, or *P* < 10^−5^ in the scan including the eight residual outliers and *P* < 10^−4^ in the primary scan. Where possible, the SNP with the smallest *P*-value in each region was replicated, along with the second best hit.

## SUPPLEMENTARY MATERIAL

Supplementary Material is available at *HMG* online.

## FUNDING

The principal funding for this study was provided by the Wellcome Trust, as part of the WTCCC2 project (grant numbers 085475/B/08/Z, 085475/Z/08/Z). C.C.A.S. is supported by a Wellcome Trust Fellowship (grant number 097364/Z/11/Z). P.D. was supported in part by a Royal Society Wolfson Merit Award and a Wellcome Trust Senior Investigator Award (095552/Z/11/Z). The work was also supported in part by Wellcome Trust Centre for Human Genetics core grants (grant numbers 090532/Z/09/Z, 075491/Z/04/B). Funding to pay the Open Access publication charges for this article was provided by the Wellcome Trust.

## Supplementary Material

Supplementary Data

## APPENDIX: LIST OF AUTHORS

The authors of this study are:

Amy Strange^1,†^, Céline Bellenguez^1,†^, Xueling Sim^2^, Robert Luben^3^, Pirro G. Hysi^4^, Wishal D. Ramdas^5,6^, Leonieke M.E. van Koolwijk^7^, Colin Freeman^1^, Matti Pirinen^1^, Zhan Su^1^, Gavin Band^1^, Richard Pearson^1^, Damjan Vukcevic^1^, Cordelia Langford^8^, Panos Deloukas^8^, Sarah Hunt ^8^, Emma Gray^8^, Serge Dronov^8^, Simon C. Potter^8^, Avazeh Tashakkori-Ghanbaria^8^, Sarah Edkins^8^, Suzannah J. Bumpstead^8^, Jenefer M. Blackwell^9,10^, Elvira Bramon^11,12^, Matthew A. Brown^13^, Juan P. Casas^14,15^, Aiden Corvin^16^, Audrey Duncanson^17^, Janusz A.Z. Jankowski^18-20^, Hugh S. Markus^21^, Christopher G. Mathew^22^, Colin N.A. Palmer^23^, Robert Plomin^24^, Anna Rautanen^1^, Stephen J. Sawcer^25^, Richard C. Trembath^22^, Nicholas W. Wood^26^, Ines Barroso^8^, Leena Peltonen^8^, Paul Healey^27^, Peter McGuffin^24^, Fotis Topouzis^28^, Caroline C.W. Klaver^5,6^, Cornelia M. van Duijn^5^, David A. Mackey^29^, Terri L. Young^30^, Christopher J. Hammond^4^, Kay-Tee Khaw^3^, Nick Wareham^31^, Jie Jin Wang^32,33^, Tien Y. Wong^34,35^, Paul J. Foster^36^, Paul Mitchell^27,33^, Chris C.A. Spencer^1^, Peter Donnelly^1,37,‡,*^ and Ananth C. Viswanathan^36,‡^

^1^Wellcome Trust Centre for Human Genetics, University of Oxford, Roosevelt Drive, Oxford OX3 7BN, UK, ^2^National University of Singapore, Singapore, ^3^Department of Public Health and Primary Care Institute of Public Health, University of Cambridge School of Clinical Medicine, Cambridge CB2 0SR, UK, ^4^Department of Twin Research and Genetic Epidemiology, King's College London, St Thomas' Hospital, UK, ^5^Department of Epidemiology and ^6^Department of Ophthalmology, Erasmus Medical Center, Rotterdam, The Netherlands, ^7^The Rotterdam Eye Hospital Glaucoma Service, Rotterdam, The Netherlands, ^8^Wellcome Trust Sanger Institute, Wellcome Trust Genome Campus, Hinxton, Cambridge CB10 1SA, UK, ^9^Telethon Institute for Child Health Research, Centre for Child Health Research, University of Western Australia, 100 Roberts Road, Subiaco, Western Australia 6008, ^10^Cambridge Institute for Medical Research, University of Cambridge School of Clinical Medicine, Cambridge CB2 0XY, UK, ^11^UCL Institute of Cognitive Neuroscience, University College London, London W1W 7EJ, UK, ^12^UCL Mental Health Sciences Unit, University College London, London W1W 7EJ, UK, ^13^University of Queensland Diamantia Institute, Princess Alexandra Hospital, University of Queensland, Brisbane, Queensland, Australia, ^14^Department of Epidemiology and Population Health London School of Hygiene and Tropical Medicine, London WC1E 7HT, UK, ^15^Department of Epidemiology and Public Health, University College London, London WC1E 6BT, UK, ^16^Neuropsychiatric Genetics Research Group, Institute of Molecular Medicine, Trinity College Dublin, Dublin 2, Ireland, ^17^Molecular and Physiological Sciences, The Wellcome Trust, London NW1 2BE, UK, ^18^Centre for Digestive Diseases, Blizard Institute, Queen Mary University of London, London E1 2AD, UK, ^19^Department of Medical Oncology, Churchill Hospital, University of Oxford, Oxford OX3 7DQ, UK, ^20^Digestive Disease Academic Centre, Leicester Royal Infirmary, Leicester LE7 7HH, UK, ^21^Clinical Neurosciences, St George's University of London, London SW17 0RE, UK, ^22^King's College London Dept Medical and Molecular Genetics, King's Health Partners Guy's Hospital, London SE1 9RT, UK, ^23^Biomedical Research Centre, Ninewells Hospital and Medical School, Dundee DD1 9SY, UK, ^24^MRC Social Genetic and Developmental Psychiatry Centre, Institute of Psychiatry, King's College London, London SE5 8AF, UK, ^25^Department of Clinical Neurosciences, University of Cambridge, Addenbrooke's Hospital, Cambridge CB2 0QQ, UK, ^26^Department of Molecular Neuroscience, Institute of Neurology, University College London, Queen Square, London WC1N 3BG, UK, ^27^Department of Ophthalmology, University of Sydney, Sydney, NSW 2006, Australia, ^28^Department of Ophthalmology, School of Medicine, Aristotle University of Thessaloniki, AHEPA Hospital, Thessaloniki, Greece, ^29^Centre for Ophthalmology and Visual Science and Lions Eye Institute, University of Western Australia, Perth, Australia, ^30^Center for Human Genetics, Duke University Medical Center, Durham NC, USA, ^31^Medical Research Council Epidemiology Unit, Cambridge CB2 0QQ, UK, ^32^Centre for Eye Research Australia (CERA), Department of Ophthalmology, University of Melbourne, Melbourne 3002, Australia, ^33^Centre for Vision Research, Department of Ophthalmology and Westmead Millenium Institute, University of Sydney, Sydney, NSW 2006, Australia, ^34^Department of Epidemiology and Public Health, National University of Singapore, Singapore, ^35^Singapore Eye Research Institute, Singapore National Eye Centre, National University of Singapore, Singapore, ^36^NIHR Biomedical Research Centre at Moorfields Eye Hospital NHS Foundation Trust and UCL Institute of Ophthalmology, London EC1V 2PD, UK, ^37^Department of Statistics, University of Oxford, Oxford OX1 3TG, UK.

†These authors contributed equally to this work.

‡These authors jointly supervised this work.
