# Supplementary material for: Genome-wide association study of intraocular pressure identifies the GLCCI1/ICA1 region as a glaucoma susceptibility locus
Source: Hum Mol Genet. 2013 Jul 7;22(22):4653–60. doi: 10.1093/hmg/ddt293 (PMC3904806; doi:10.1093/hmg/ddt293)
Supplement: Supplementary Data [file supp_22_22_4653__index.html]

Genome-wide association study of intraocular pressure identifies the GLCCI1/ICA1 region as a glaucoma susceptibility locus — Genome-wide association study of intraocular pressure identifies the GLCCI1/ICA1 region as a glaucoma susceptibility locus — Genome-wide association study of intraocular pressure identifies the GLCCI1/ICA1 region as a glaucoma susceptibility locus — Supplementary Data 

# Genome-wide association study of intraocular pressure identifies the GLCCI1/ICA1 region as a glaucoma susceptibility locus

## 

Supplementary Data

**Files in this Data Supplement:**

- Supplementary Data - Doc file
- WTCCC2\_IOP\_summary - txt file
