# Supplementary material for: Genome-wide association study of intraocular pressure identifies the GLCCI1/ICA1 region as a glaucoma susceptibility locus
Source: Hum Mol Genet. 2013 Jul 7;22(22):4653–60. doi: 10.1093/hmg/ddt293 (PMC3904806; doi:10.1093/hmg/ddt293)
Supplement: Supplementary Data [file supp_ddt293_ddt293supp.doc]

GL Supplementary

**SUPPLEMENTAL INFORMATION**

**Membership of The Blue Mountains Eye Study (BMES) GWAS Team**

Jie Jin Wang^1,2^, Paul Mitchell^2^, Ananth C Viswanathan^3^, Tien Y Wong^1,4^, Elena Rochtchina^2^, Jing Xie^1^, Xueling Sim^5^, Michael Inouye^6^, Elizabeth G Holliday^7,8^, John Attia^7,8^, Rodney J Scott^8,9,10^, Paul N Baird^1^

1Centre for Eye Research Australia, University of Melbourne, Royal Victorian Eye and Ear Hospital, Melbourne, Australia; 2Centre for Vision Research, Department of Ophthalmology and Westmead Millennium Institute, University of Sydney, NSW Australia; 3NIHR Biomedical Research Centre at Moorfields Eye Hospital NHS Foundation Trust and UCL Institute of Ophthalmology; 4Singapore Eye Research Institute, Singapore National Eye Centre, National University of Singapore, Singapore; 5National University of Singapore, Singapore; 6The Walter and Elisa Hall Institute of Medical Research, Victoria, Australia; 7School of Medicine and Public Health, University of Newcastle, Newcastle, Australia; 8Hunter Medical Research Institute, Newcastle, Australia; 9The Centre for Information Based Medicine and the School of Biomedical Sciences and Pharmacy University of Newcastle, Newcastle, Australia; 10The Division of Genetics, Hunter Area Pathology Service, John Hunter Hospital, Newcastle, Australia.

**Membership of Wellcome Trust Case Control Consortium 2**

Management Committee

Peter Donnelly (Chair)^1,2^, Ines Barroso (Deputy Chair)^3^, Jenefer M Blackwell^4, 5^, Elvira Bramon^6^ , Matthew A Brown^7^ , Juan P Casas^8^ , Aiden Corvin^9^, Panos Deloukas^3^, Audrey Duncanson^10^, Janusz Jankowski^11^, Hugh S Markus^12^, Christopher G Mathew^13^, Colin NA Palmer^14^, Robert Plomin^15^, Anna Rautanen^1^, Stephen J Sawcer^16^, Richard C Trembath^13^, Ananth C Viswanathan^17^, Nicholas W Wood^18^

Data and Analysis Group

Chris C A Spencer^1^, Gavin Band^1^, Céline Bellenguez^1^, Colin Freeman^1^, Garrett Hellenthal^1^, Eleni Giannoulatou^1^, Matti Pirinen^1^, Richard Pearson^1^, Amy Strange^1^, Zhan Su^1^, Damjan Vukcevic^1^, Peter Donnelly^1,2^

DNA, Genotyping, Data QC and Informatics Group

Cordelia Langford^3^, Sarah E Hunt^3^, Sarah Edkins^3^, Rhian Gwilliam^3^, Hannah Blackburn^3^, Suzannah J Bumpstead^3^, Serge Dronov^3^, Matthew Gillman^3^, Emma Gray^3^, Naomi Hammond^3^, Alagurevathi Jayakumar^3^, Owen T McCann^3^, Jennifer Liddle^3^, Simon C Potter^3^, Radhi Ravindrarajah^3^, Michelle Ricketts^3^, Avazeh Tashakkori-Ghanbaria^3^, Matthew Waller^3^, Paul Weston^3^, Sara Widaa^3^, Pamela Whittaker^3^, Ines Barroso^3^, Panos Deloukas^3^**.**

Publications Committee

Christopher G Mathew (Chair)^13^, Jenefer M Blackwell^4,5^, Matthew A Brown^7^, Aiden Corvin^9^, Mark I McCarthy^19^, Chris C A Spencer^1^

1 Wellcome Trust Centre for Human Genetics, Roosevelt Drive, Oxford OX3 7BN, UK; 2 Dept Statistics, University of Oxford, Oxford OX1 3TG, UK; 3 Wellcome Trust Sanger Institute, Wellcome Trust Genome Campus, Hinxton, Cambridge CB10 1SA, UK; 4 Telethon Institute for Child Health Research, Centre for Child Health Research, University of Western Australia, 100 Roberts Road, Subiaco, Western Australia 6008; 5 Cambridge Institute for Medical Research, University of Cambridge School of Clinical Medicine, Cambridge CB2 0XY, UK; 6 UCL Mental Health Sciences Unit and UCL Institute of Cognitive Neuroscience, University College London, London W1W 7EJ, UK; 7 Diamantina Institute of Cancer, Immunology and Metabolic Medicine, Princess Alexandra Hospital, University of Queensland, Brisbane, Queensland, Australia; 8 Dept Epidemiology and Population Health, London School of Hygiene and Tropical Medicine, London WC1E 7HT and Dept Epidemiology and Public Health, University College London WC1E 6BT, UK; 9 Neuropsychiatric Genetics Research Group, Institute of Molecular Medicine, Trinity College Dublin, Dublin 2, Ireland; 10 Molecular and Physiological Sciences, The Wellcome Trust, London NW1 2BE; 11 Centre for Digestive Diseases, Queen Mary University of London, London E1 2AD, UK and Digestive Diseases Centre, Leicester Royal Infirmary, Leicester LE7 7HH, UK and Department of Clinical Pharmacology, Old Road Campus, University of Oxford, Oxford OX3 7DQ, UK; 12 Clinical Neurosciences, St George's University of London, London SW17 0RE; 13 King’s College London Dept Medical and Molecular Genetics, School of Medicine, Guy’s Hospital, London SE1 9RT, UK; 14 Biomedical Research Centre, Ninewells Hospital and Medical School, Dundee DD1 9SY, UK; 15 King’s College London Social, Genetic and Developmental Psychiatry Centre, Institute of Psychiatry; 16 University of Cambridge Dept Clinical Neurosciences, Addenbrooke’s Hospital, Cambridge CB2 0QQ, UK; 17 NIHR Biomedical Research Centre at Moorfields Eye Hospital NHS Foundation Trust and UCL Institute of Ophthalmology, London EC1V 2PD, UK; 18 Dept Molecular Neuroscience, Institute of Neurology, Queen Square, London WC1N 3BG, UK; 19 Oxford Centre for Diabetes, Endocrinology and Metabolism (ICDEM), Churchill Hospital, Oxford OX3 7LJ, UK.

**Membership of EPIC Eyes group**

Cambridge- Institute of Public Health ([http://www.iph.cam.ac.uk](https://amsprd0104.outlook.com/owa/redir.aspx?C=_IOcPJFS70SNoNtzuwwnWOZpBDOUJs8IIl6OVX4YLcfd3A2w-AqGatrt5xc34wHBpwJt9wC5izg.&URL=http%3A%2F%2Fwww.iph.cam.ac.uk)/)

Kay-Tee Khaw, Shabina Hayat, Robert Luben, Nichola Dalzell, Anthony Khawaja, Jennifer Yip, Stephanie Moore, Elliot Grigg, Amit Bhaniani

Cambridge- MRC Epidemiology Unit ([http://www.mrc-epid.cam.ac.uk](https://amsprd0104.outlook.com/owa/redir.aspx?C=_IOcPJFS70SNoNtzuwwnWOZpBDOUJs8IIl6OVX4YLcfd3A2w-AqGatrt5xc34wHBpwJt9wC5izg.&URL=http%3A%2F%2Fwww.mrc-epid.cam.ac.uk)/)

Nick Wareham

Cambridge- Department of Ophthalmology

Keith Martin, Humma Shahid

Norfolk & Norwich University Hospital NHS Foundation Trust

David Broadway, Heidi Cate, Annie Few

UCL- IOO Division of Genetics and Epidemiology

Paul J Foster, Michelle Chan, Pak Sang Lee, Alex Day

**SNP Imputation**. All imputation for the discovery phase was performed with IMPUTE2, using the 1000 Genomes, as the reference panel. Prior to imputation SNPs were filtered using the following thresholds: <0.1% minor allele frequency, Hardy Weinberg P<10^-20^, info <0.975 and >2% missing data. IMPUTE1 was run before IMPUTE2 to identify SNPs which imputed poorly but with high confidence. This was determined by the difference between info and the concordance score outputted by IMPUTE1. The concordance measure is taken at SNPs for which genotype data is available, where concordance between the imputed calls and the genotyped calls are measured. Calculating the difference between info and concordance is a measure of the imputation accuracy with respect to the confidence, and SNPs were removed if this difference was >0.05.

**SUPPLEMENTARY FIGURES**

**
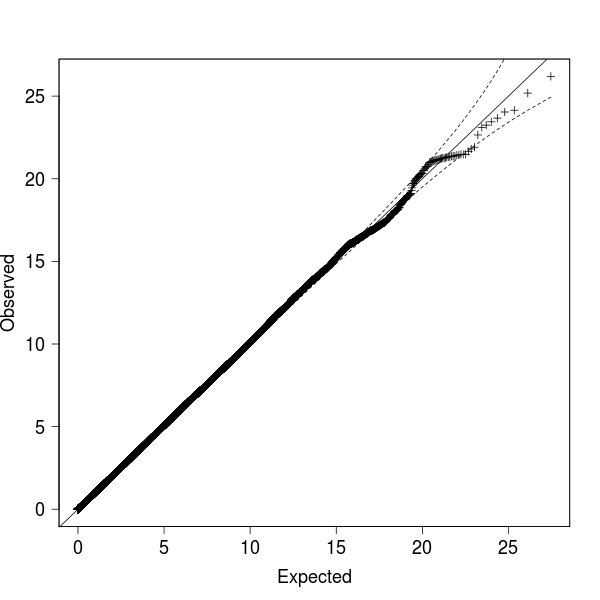
**

**Supplementary Figure 1** Quantile-quantile plot showing the distribution of the test statistic of autosomal SNPs (genotyped and imputed) after quality control. The ratio of the observed to expected media test statistic (lambda) is 1.01. The dotted lines show the 95% confidence interval.

**
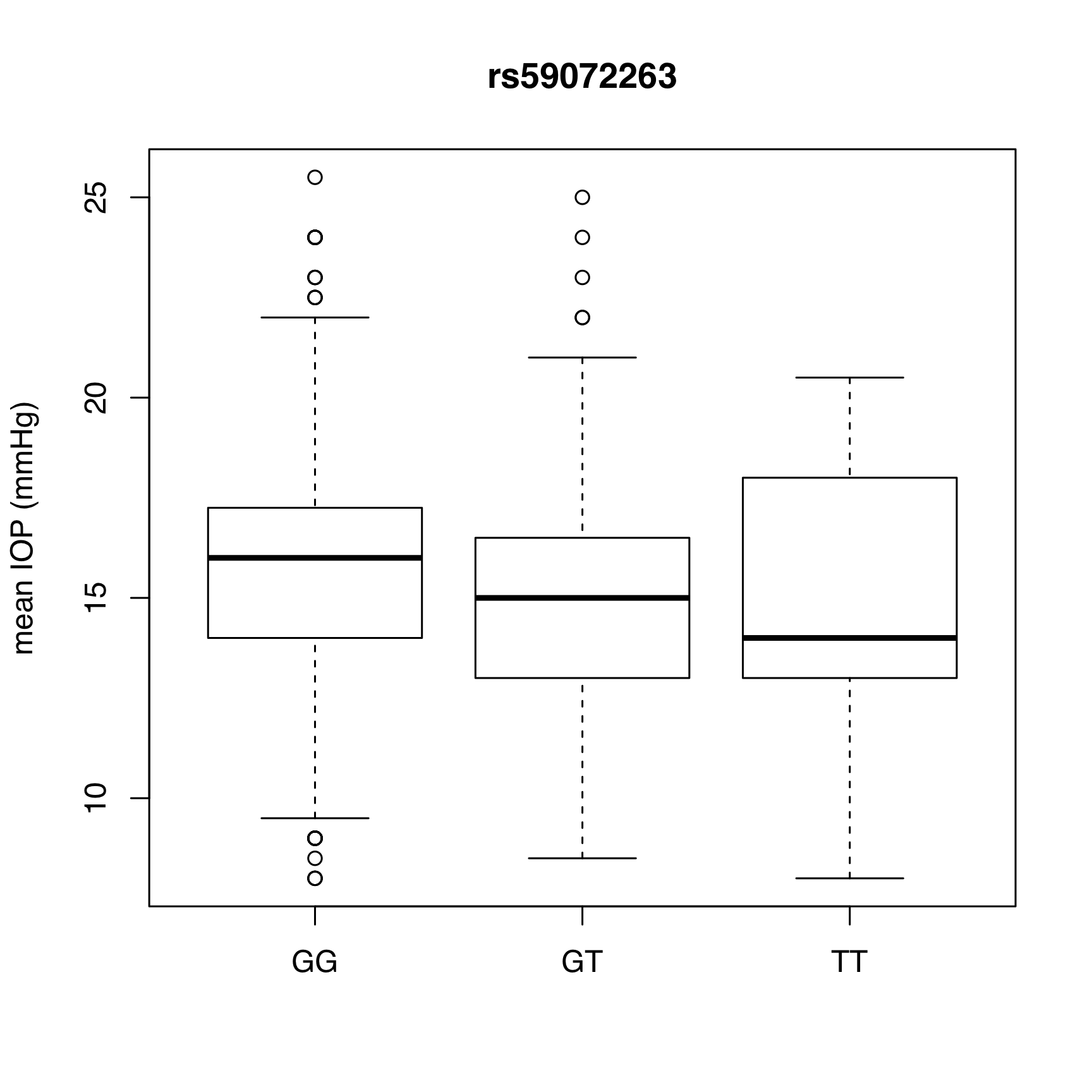
**

**Supplementary Figure 2** Boxplot of the mean IOP and genotype at rs59072263 in the discovery data.

**
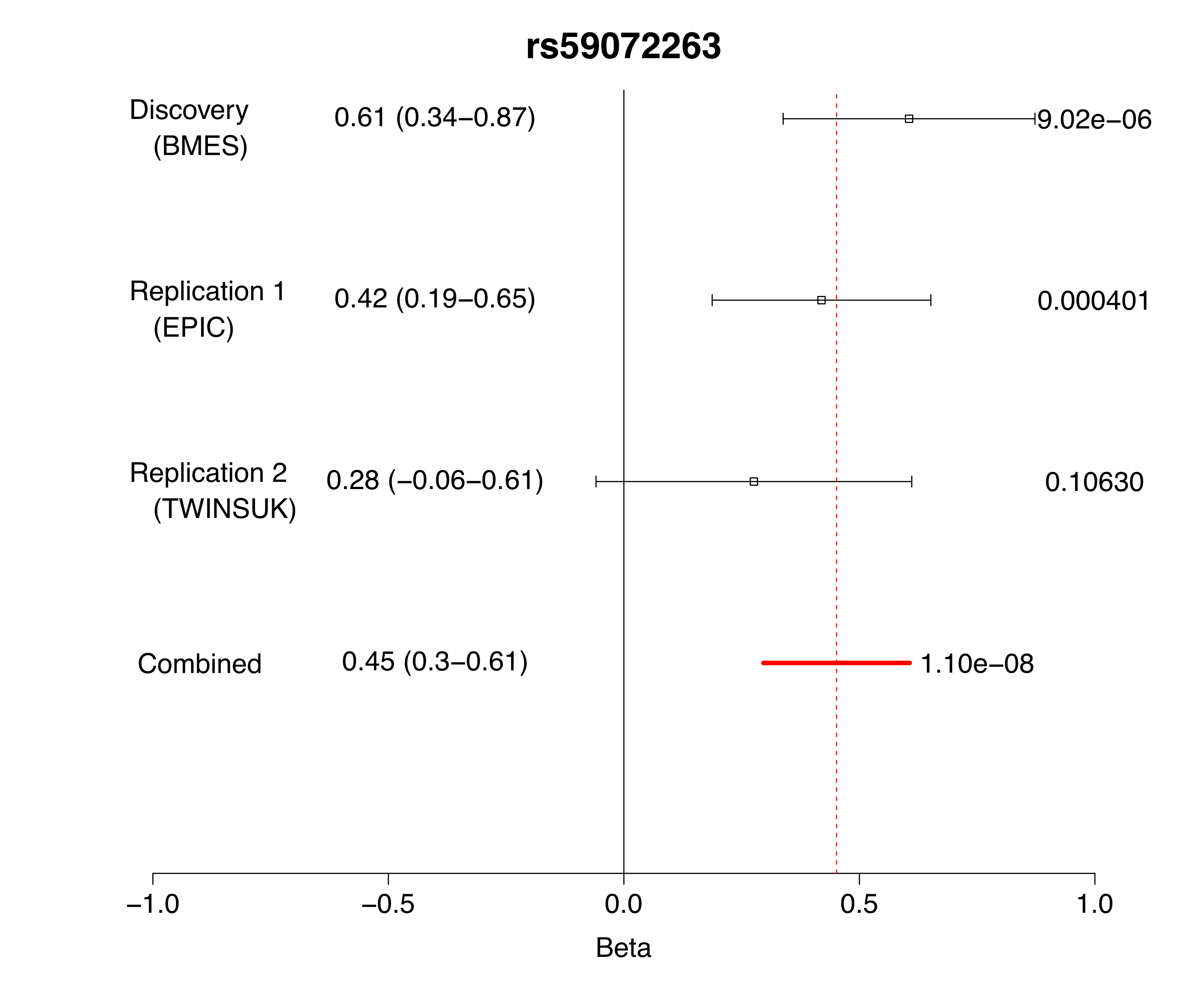
**

**Supplementary Figure 3** Forest plot of rs59072263 on chromosome 7p21. The effect size is given on the left, next to the study label, and the P-value for the study is shown on the right.

SUPPLEMENTARY TABLE

|  | | | | Discovery | | | | | Replication | | | Meta |
| --- | --- | --- | --- | --- | --- | --- | --- | --- | --- | --- | --- | --- |
| Chr | rsID | Position | RA | RAF | P value | Beta | SE | G/I | P value | Beta | SE | P value |
| 1 | rs10917773 | 161716613 | T | 0.44 | 2.37E-05 | 0.365 | 0.086 | 1 | 0.631 | 0.041 | 0.085 | 9.32E-04 |
| 1 | rs7555523^a^ | 163985603 | C | 0.12 | 0.030 | 0.268 | 0.124 | 1 | 1.70E-03 | 0.408 | 0.130 | 1.88E-04 |
| 1 | rs7518099^a^ | 164003504 | C | 0.12 | 0.030 | 0.268 | 0.124 | 2 | 7.97E-04 | 0.441 | 0.131 | 1.04E-04 |
| 1 | rs1987944 | 169458580 | C | 0.85 | 1.54E-06 | 0.558 | 0.116 | 1 | 0.696 | -0.047 | 0.119 | 1.51E-03 |
| 1 | rs12065544 | 169459653 | A | 0.85 | 1.43E-06 | 0.560 | 0.116 | 1 | 0.601 | -0.063 | 0.120 | 1.91E-03 |
| 4 | rs16837723 | 5892251 | G | 0.13 | 4.25E-06 | 0.565 | 0.123 | 2 | 0.238^d^ | 0.156 | 0.132 | 3.05E-05 |
| 6 | rs62408171 | 90709616 | G | 0.81 | 3.91E-06 | 0.489 | 0.106 | 1 | 0.572 | -0.064 | 0.113 | 2.87E-03 |
| 6 | rs56405757 | 90709750 | C | 0.81 | 2.86E-06 | 0.500 | 0.107 | 1 | 0.665 | -0.049 | 0.113 | 1.89E-03 |
| 7 | rs13227590 | 8117515 | G | 0.82 | 3.91E-05 | 0.460 | 0.112 | 1 | 5.44E-04 | 0.397 | 0.115 | 8.25E-08 |
| 7 | rs59072263 | 8118592 | G | 0.88 | 9.02E-06 | 0.605 | 0.136 | 1 | 4.01E-04 | 0.420 | 0.118 | 2.32E-08 |
| 9 | rs17370022^b^ | 35414038 | A | 0.94 | 3.24E-07 | 2.678 | 0.532 | 1 | 0.853 | -0.103 | 0.554 | 4.56E-04 |
| 9 | rs17298052^b^ | 35428294 | G | 0.94 | 3.38E-07 | 2.674 | 0.531 | 1 | 0.852 | -0.103 | 0.554 | 4.56E-04 |
| 9 | rs17370251^b^ | 35429386 | T | 0.94 | 3.06E-07 | 2.693 | 0.532 | 1 | 0.851 | -0.104 | 0.554 | 4.56E-04 |
| 10 | rs2578121 | 79887214 | C | 0.41 | 1.49E-05 | 0.362 | 0.084 | 1 | 0.692 | -0.034 | 0.085 | 4.94E-03 |
| 10 | rs7918709 | 79887974 | G | 0.47 | 1.94E-06 | 0.392 | 0.082 | 1 | 0.394 | -0.072 | 0.085 | 4.83E-03 |
| 10 | rs7094656 | 79893524 | G | 0.26 | 1.51E-05 | 0.417 | 0.096 | 1 | 0.807 | -0.024 | 0.096 | 3.88E-03 |
| 10 | rs3736580^c^ | 124739955 | T | 0.10 | 1.47E-05 | 0.600 | 0.138 | 1 | 0.280^d^ | 0.150 | 0.139 | 1.26E-04 |
| 10 | rs9423302^c^ | 124743050 | A | 0.09 | 2.05E-05 | 0.590 | 0.138 | 1 | 0.440 | 0.104 | 0.135 | 4.25E-04 |
| 11 | rs1908161^c^ | 25657046 | G | 0.07 | 1.26E-04 | 0.619 | 0.162 | 1 | 0.066 | -0.334 | 0.181 | 0.101 |
| 11 | rs12292495 | 38715491 | A | 0.55 | 9.49E-06 | 0.379 | 0.085 | 1 | 0.061 | -0.158 | 0.085 | 0.074 |
| 11 | rs10430986 | 41139095 | C | 0.04 | 3.02E-06 | 0.935 | 0.200 | 1 | 0.070 | 0.410 | 0.226 | 2.63E-06 |
| 11 | rs12222492 | 41144435 | T | 0.04 | 3.58E-06 | 0.933 | 0.201 | 1 | 0.113 | 0.334 | 0.211 | 8.68E-06 |
| 11 | rs1783582^c^ | 72569148 | A | 0.45 | 1.39E-04 | 0.315 | 0.083 | 1 | 0.741 | 0.028 | 0.085 | 3.11E-03 |
| 11 | rs947848^c^ | 72572655 | T | 0.45 | 7.74E-05 | 0.369 | 0.093 | 1 | 0.868 | 0.014 | 0.084 | 5.55E-03 |
| 13 | rs17798800 | 33848527 | T | 0.20 | 3.26E-05 | 0.429 | 0.103 | 2 | 0.852 | 0.020 | 0.107 | 1.82E-03 |
| 14 | rs3818077 | 101048522 | A | 0.54 | 8.93E-07 | 0.405 | 0.082 | 1 | 0.713 | 0.032 | 0.086 | 1.41E-04 |
| 14 | rs3818079 | 101049041 | G | 0.53 | 5.22E-07 | 0.411 | 0.082 | 2 | 0.316^d^ | 0.092 | 0.092 | 9.99E-06 |
| 17 | rs28391220^a^ | 9972548 | C | 0.54 | 0.492 | 0.060 | 0.088 | 1 | 5.39E-03 | 0.238 | 0.085 | 0.013 |
| 17 | rs11656696^a^ | 9974404 | C | 0.58 | 0.548 | 0.051 | 0.084 | 2 | 0.016 | 0.206 | 0.085 | 0.034 |
| 17 | rs9910961^c^ | 73341718 | T | 0.40 | 4.18E-05 | 0.343 | 0.084 | 2 | 0.879^d^ | -0.014 | 0.090 | 3.81E-03 |
| 17 | rs9911139 ^c^ | 73341761 | T | 0.40 | 4.44E-05 | 0.342 | 0.084 | 1 | 0.913 | 0.009 | 0.084 | 2.94E-03 |

**Supplementary Table 1.** All SNPs for which a first round of replication was attempted.

RA – Risk allele, RAF – risk allele frequency. G/I shows whether the SNP was imputed (1) or genotyped (2) in the discovery data. ^a^SNPs chosen to replicate previously identified associated loci, ^b^SNP found to be associated using a general model with residual outliers included, therefore results are presented at this SNP for this analysis with the additive Beta shown, ^c^SNPs found to be associated in the scan which included phenotype residual outliers, therefore results are presented at this SNP for this analysis, ^d^Replication was attempted using data from the Immunochip array, rather than from the Sequenom plex.
